# Cerebral fat embolism after traumatic bone fractures: a structured literature review and analysis of published case reports

**DOI:** 10.1186/s13049-021-00861-x

**Published:** 2021-03-12

**Authors:** Luigi Vetrugno, Elena Bignami, Cristian Deana, Flavio Bassi, Maria Vargas, Maria Orsaria, Daniele Bagatto, Cristina Intermite, Francesco Meroi, Francesco Saglietti, Marco Sartori, Daniele Orso, Massimo Robiony, Tiziana Bove

**Affiliations:** 1 Department of Anesthesia and Intensive Care, Anesthesia and Intensive Care Clinic, Azienda Sanitaria Universitaria Friuli Centrale, Udine, Italy; 2 Department of Medicine, Anesthesia and Intensive Care Clinic, University of Udine, Udine, Italy; 3 Department of Medicine and Surgery, Unit of Anesthesiology, Parma University Hospital, Parma, Italy; 4 Department of Anesthesia and Intensive Care, Anesthesia and Intensive Care Unit 1, Azienda Sanitaria Universitaria Friuli Centrale, Udine, Italy; 5 Department of Anesthesia and Intensive Care, Anesthesia and Intensive Care Unit 2, Azienda Sanitaria Universitaria Friuli Centrale, Udine, Italy; 6 Department of Neurosciences, Reproductive and Odontostomatological Sciences, University of Naples “Federico II”, Naples, Italy; 7 Department of Medicine, Surgical Pathology Section, University of Udine, Udine, Italy; 8 Department of Diagnostic Imaging, Neuroradiology Unit, Azienda Sanitaria Universitaria Friuli Centrale, Udine, Italy; 9 School of Medicine and Surgery, University of Milan-Bicocca, Monza, Italy; 10 Department of Medicine, Maxillofacial Surgery, University of Udine, Udine, Italy; 11 Azienda Sanitaria Universitaria Friuli Centrale, Maxillofacial Surgery, Udine, Italy

**Keywords:** Cerebral fat embolism, Fat embolism syndrome, Patent foramen ovale

## Abstract

**Background:**

The incidence of cerebral fat embolism (CFE) ranges from 0.9–11%, with a mean mortality rate of around 10%. Although no univocal explanation has been identified for the resulting fat embolism syndrome (FES), two hypotheses are widely thought: the ‘mechanical theory’, and the ‘chemical theory’. The present article provides a systematic review of published case reports of FES following a bone fracture.

**Methods:**

We searched MEDLINE, Web of Science and Scopus to find any article related to FES. Inclusion criteria were: trauma patients; age ***≥*** 18 years; and the clinical diagnosis of CFE or FES. Studies were excluded if the bone fracture site was not specified.

**Results:**

One hundred and seventy studies were included (268 cases). The male gender was most prominent (81.6% vs. 18.4%). The average age was 33 years (±18). The mean age for males (29 ± 14) was significantly lower than for females (51 ± 26) (*p* < 0.001). The femur was the most common fracture site (71% of cases). PFO was found in 12% of all cases. Univariate and multivariate regression analyses showed the male gender to be a risk factor for FES: RR 1.87 and 1.41, respectively (95%CI 1.27–2.48, *p* < 0.001; 95%CI 0.48–2.34, *p* < 0.001).

**Conclusions:**

FES is most frequent in young men in the third decades of life following multiple leg fractures. FES may be more frequent after a burst fracture. The presence of PFO may be responsible for the acute presentation of cerebral embolisms, whereas FES is mostly delayed by 48–72 h.

**Supplementary Information:**

The online version contains supplementary material available at 10.1186/s13049-021-00861-x.

## Introduction

**Fig. 1:**
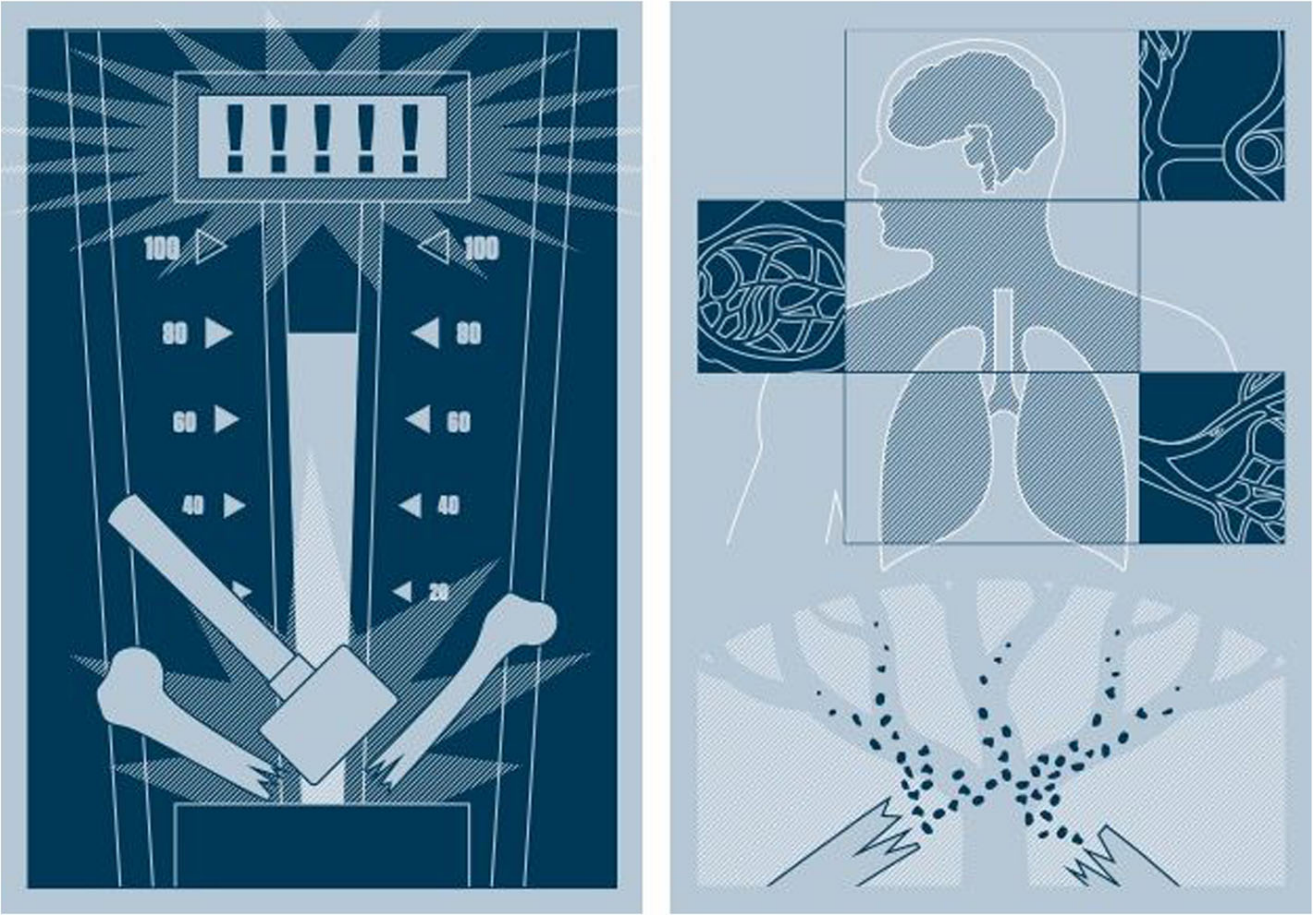
A simple model based on a fairground ‘test your strength’ hammer game might explain why higher energy impacts are more likely to result in fat emboli being ‘shot’ into the vascular compartment able to reach the upper body. Multiple organ involvement of fat embolization according to Peltier’s mechanical theory

Cerebral fat embolism (CFE) is a rare and potentially fatal condition that may occur following a long bone fracture or pelvis trauma, showing an incidence ranging from 0.9 to 11% with a mean mortality rate around 10% [1–3]. After a ‘bone burst’, according to the accepted pathophysiology, ‘fat droplets’ are shot into the systemic circulation, giving rise to emboli [4, 5]. But no univocal explanation exists to describe how the syndrome develops from hereon. Two hypotheses have been validated to account for the early and late symptoms, respectively. The “mechanical theory” describes marrow fat being pushed into the veins as a result of the increased intramedullary pressure, generating respiratory and neurological changes and the emergence of non-palpable skin petechiae in the upper body area (axillae, trunk and sclera) 2 or 3 days following the trauma [6, 7]. This triad of symptoms is known as fat embolism syndrome (FES). The most common sub-clinical presentation of FES can be reasonably identified as a reduction in respiratory gas-exchange and petechiae [8, 9], whereas the most severe “mechanical” scenario results in acute respiratory symptoms, acute right heart failure (Peltier’s theory) [10] and even abrupt brain death. The “chemical theory” is built on evidence showing that products of the systemic inflammatory response, secondary to the initial bone trauma, trigger the conversion of fat molecules into free fatty acids and glycerol that can, in turn, lead to vascular lesions [11, 12]. This chemical theory is able to account for the vascular endothelial symptoms of the affected region and explains the late signs of central nervous system damage: confusion, hemiplegia, apraxia, aphasia and lethargy. The characteristic “starry night” magnetic resonance image (MRI) of the brain appears approx. One week after the trauma [13]. In the first case, the most accredited events are: high-velocity trauma; the deferral of surgery for more than 10–24 h; multiple fractures; and, as co-factor, difficulties in fracture reduction and nailing or external fixation [14]. The potential correlation between the presence of patent foramen ovale (PFO) and FES in the latter case has yet to be explored [15, 16]. In the absence of a right-to-left shunt, fat globules would have to be small and multiple to generate a neurological presentation [17]. On the other hand, once tiny fat particles have traversed the pulmonary capillary bed, they can then reach the brain. To the best of our knowledge, the significance of a PFO has never been formally reviewed in the context of FES. Nevertheless, we suggest likening the force and result of the trauma to a fairground “test your strength” hammer game (Fig. 1). The more ‘favorable’ mechanical scenario depends on the low energy of the fracture-causing ‘strike’ in which the resulting fat embolism is not ‘strong enough’ to trigger cerebral fat embolism CFE or FES. By contrast, a more ‘critical’ mechanical scenario would be one in which a high energy blow results in the fat being ‘shot’ as far as the pulmonary capillary bed or central nervous system.

The recent literature highlights how FES has gathered recognition thanks to the clinical descriptions in case reports and case series [18–22]. However, the frequency of the syndrome is too low to permit any conclusions to be drawn from the individual studies reported in literature. Adopting a structured literature review approach, we analyzed all the relevant studies available in the literature. We aimed to provide a review of CFE occurring after a bone fracture, combined with separate examinations of published cases in adult patients. Specifically, we focused attention on identifying the associated risk factors, the laterality of the fractures, the presence of PFO, any cerebral involvement and the pathophysiology of CFE.

## Materials and methods

### Article selection

We conducted a literature analysis between 8th October 2018 and 8th December 2018, applying the following search terms: “cerebral fat embolism AND fat embolism syndrome”, “cerebral fat embolism OR fat embolism syndrome”, “cerebral fat embolism OR fat embolism syndrome AND case report” and “cerebral fat embolism AND fat embolism syndrome AND case report” to search the MEDLINE (1960–2018), SCOPUS (1960–2018) and WEB OF SCIENCES (1960–2018) databases. Peer-reviewed studies written in English and presumed to report human cases of CFE in adult patients were selected based upon the Preferred Reporting Items for Systematic Review and Meta-Analyses (PRISMA) protocol [23]. This systematic review was formerly registered in the prospective international register of systematic reviews – PROSPERO – date: 30th January 2019 – with the identification number #CRD42019114334.

### Data collecting and handling

All articles were selected, screened and reviewed, and the data from each case report collated and registered into a Microsoft Excel (Microsoft, Redmond, Washington, USA) spreadsheet. The data included: age; sex; body mass index (BMI); the site, number and side (right or left or bilateral) of bone fracture; the presence of PFO; and the location and side of cerebral involvement diagnosed with computed tomography (CT) or a magnetic resonance image (MRI) brain scan. In the case that some features were absent, data were coded as ‘missing’.

### Study selection and eligibility criteria

Inclusion criteria were: trauma patients; age ≥ 18 years; and clinical diagnosis of CFE or FES. Studies were excluded in the absence of data about the bone fracture site. Five authors (C.I., F. M, F. S, C.D. and M.S.) carried out two independent searches to identify eligible studies for the pooled analysis. The data retrieved by the researchers from these studies were compared. The eligibility of each study was decided by all five researchers. We established a priori that all disputes would be resolved through the consultation of the senior investigator (L.V.).

### Statistical analysis

The following data were collected from each study: sex; age; presence of PFO patency; presence of a brain CT scan; presence of a brain nuclear magnetic resonance image; localization of brain lesions; and localization of various fractures. Data were reported as means ± standard deviations (S.D.), medians and interquartile ranges, or proportions as appropriate. Comparisons between groups were performed using the Mann-Whitney U test or two-way ANOVA for continuous variables, and Fisher’s test or the χ^2^ test for categorical variables. For the analysis, we divided patients according to sex and age. Univariate logistic regression was performed to identify clinically essential variables potentially associated with CFE between males and females and between different ages. Multivariate logistic regressions were carried out using backward stepwise variable elimination and included the variables that reached a statistical significance at the univariate logistic regression. Statistical significance was set at α = 0.05. Statistical analysis was performed using SPSS (version 20.0, IBM®).

## Results

We identified a total of 170 research studies for review as depicted in the PRISMA flow diagram (Figure S1 on Supplementary Material), providing a total of 268 cases of FES (Table S1 on Supplementary Material). As expected, gender and age were dominant risk factors, with males more at risk than females (81.6% vs. 18.4%). The average age of all patients was 33 years (± 18), whereas the mean age of males was significantly lower than that for females, at 29 (±14) versus 51 (±26) years, respectively (*p* < 0.001), despite a spectrum of diverse attitude and frequency in the two classes (Table S2 on Supplementary Material). In particular, the age category containing the highest proportion of male cases was 21–30 years, whereas female cases were more equally distributed between age categories, although the highest proportion fell into the 71–80 age range. In the preliminary analysis there were a very small number of traumatic pediatric cases: 8 cases in total aged < 18 years (1 case 13 years, 1 case 15 years, 1 case 16 years, 5 cases 17 years). A bar chart demonstrating the distribution for each gender according to age is presented in Figure S2 (supplementary material) .

Regarding to fracture site, 71% of cases involved the femur, 36% the tibia, and 19% the fibula (isolated or crus fracture). Other fracture sites (e.g. radius, ulna, ribs) exhibited incidences of less than 10% (Table S3 on Supplementary Material). The fractures were more often bilateral in males, with higher involvement of the left side compared with females (21.4 vs 4.2; *p* = 0.031). However, the right side was the most frequently involved in both sexes (Table S4 on Supplementary Material). Examining the fracture sites according to age category, lower limb fractures constitute the most common fractures in all age groups, whereas fractures of the upper limbs were most common in the between 19 to 30 age group. Fractures of the chest wall were most prevailing between 51 and 60 years (Table S5 on Supplementary Material). Concerning the presentation of FES as confirmed by CT or MR imaging (Table S6 on Supplementary Material), the most involved sites were the internal capsule (82.4%) and subcortical zones (89%). The most affected brain lobes were the frontal (31.7%) and parietal lobes (25.4%). In 12.2% of cases, more than three brain regions were affected simultaneously (Table S6 on Supplementary Material). Pathological changes in the brain, as identified by CT imaging, were found in 73% of cases, and were more frequent in males than in females (54% vs. 17%, *p* = 0.006) (Table S2 on Supplementary Material). In males, the involvement of the temporal lobes was often bilateral (*p* = 0.005); in females, it was mostly unilateral (Table S7 on Supplementary Material). CT scans positive for fat emboli revealed a main peak in the age category 19 to 30 years (33% of cases) and a second minor peak (9.5% of cases) in those aged over 70 years of age. By contrast, the pathological findings as revealed by NMR seem to show a trimodal distribution as opposed to the two main peaks disclosed by CT scans: 32% of cases in the 19–30 years age group; 13% of cases in the 51–60 age group; and 11% of cases aged over 70 years (Table S8 on Supplementary Material). All brain regions were affected the most in the 19–30 age category, whereas the occipital lobes were least affected in all ages (Table S9 on Supplementary Material). With regard to PFO, approx. 12% of all cases were positive (8.5% men and 5.6% women, p. 0.47) – a lower incidence than reported for the whole population (25%) [24]. In particular, PFO was present in 6% of cases in the 19–30 years age group and 9% of cases over 70 years of age. Univariate logistic regression revealed both the male gender and the presence of rib fractures to constitute significant risk factors for FES (RR 1.874, 95% CI 1.272–2.477, *p* < 0.001; and RR 0.202, 95% CI 0.015–0.388, *p* = 0.034, respectively). On the other hand, multivariate logistic regression analysis revealed the male gender to be significantly associated (RR 1.412, 95% CI 0.484–2.341, *p* < 0.001), but not the presence of rib fractures (Table S10 on Supplementary Material).

Univariate logistic regression to assess risk factors for the development of FES in females compared with males showed age to be a correlated factor (RR 1.054, 95% CI 1.036–1.00, *p* < 0.001) (Table S11 on Supplementary Material). Finally, it should be noted that the number of case reports published on FES has grown considerably over the last decade (2008–2018) (Figure S3, supplemetary material).

## Discussion

The first case of fat embolism was described in 1863 by Bergmann [25]. The report regarded a patient who fell from a roof and sustained a comminuted fracture of the distal femur and developed dyspnea, cyanosis, and then fell into a coma 60 h later [25]. The patient’s autopsy revealed a massive fat embolism. The syndrome resulting from a CFE had been described just the year previous by the pathologist Friedrich Albert von Zenker [26].

In the present study, by means of PRISMA analysis, we identified 170 case report studies on CFE published in the medical literature over the last 58 years (Figure S3). Data related to 260 individual cases were extracted from these papers and subjected to meticulous statistical analysis. Table 1 on Supplementary Material summarizes the general clinical features associated with fat embolism. The data were tested for correlations between FE/FES and fracture laterality and type, the presence of PFO and the CFE physiopathology. The majority of patients affected by FE/FES were found to be young males negative for a PFO. Although the clinical significance and relevance of the results are not always very clear, especially in relation to the laterality of the fracture, a left-sided fracture was more frequent in men compared with women, although fractures were more frequent in both sexes on the right side compared with left sided or bilateral fractures.

A femoral fracture was present in 71% of cases. We do not know whether these statistics are linked to the fact that men are more likely to be involved in road traffic accidents than women.

Our data reveal the incidence of FE/FES to be higher in bilateral femoral fractures and following intramedullary nail fixation, i.e. in the long bones of the lower limbs. Multivariate analysis revealed men to be affected by FES syndrome more frequently than women. We also reveal evidence for the percentage of patients positive for FES to drop significantly after 30 years of age, although a trend for a slight rise in the number of patients was observed in those aged 51–60 years (associated with the use of MR imaging) and over 70 years. The strong reduction in the frequency of FES after 30 years of age may signify that the energy of the impacts causing bone fractures decreases as the age of the subject increases. This supports previous reports suggesting that FES is more frequently associated with complicated trauma [27–30].

Indeed, the main finding of the analysis was a correlation between gender (male), age (being younger), NMRI findings and FES presentation. On the contrary, this correlation could explain why the degree of fat embolism is sometimes not enough to trigger FES or CFE.

Although a correlation was identified between a left-sided fracture and being male, it is unclear whether a causal relationship exists or whether it is just a statistical verdict.

Acute respiratory failure after trauma may arise due to a number of causes; for example, pneumothorax, hemothorax, airway aspiration, lung contusion or acute pulmonary embolism [30, 31]. In this last scenario, a fat embolism can cause direct obstruction of the main branches of the pulmonary capillaries with acute right heart failure (Peltier’s theory) [32]. Some papers advocate the use of thrombus-pulmonary-embolectomy; however, this procedure requires specialized cardiac centers and the potential use of extracorporeal life support [33–35]. Moreover, this latter treatment, due to the necessity of heparinization constitutes a relative contraindication [33].

Non-fulminant acute pulmonary embolism is the most frequent consequence of fat embolism (FE) and it shows a different degree of respiratory failure, usually with low gas exchange, secondary to ventilation-perfusion mismatch [30].

The “late” symptoms of FES, manifesting as adult respiratory distress syndrome (ARDS), occur 2–3 days after the bone trauma (48–72 h) and are thought to be triggered by biochemical cascades following alveolar wall damage, resulting in the liberation of free fatty acids from the lysis of triglycerides [36]. The only treatment for ARDS is patient support with non-invasive or invasive artificial mechanical ventilation, depending on the gravity. In the case of invasive mechanical ventilation, a lung protective strategy has been shown to improve the outcome in these patients [37].

**Fig. 2:**
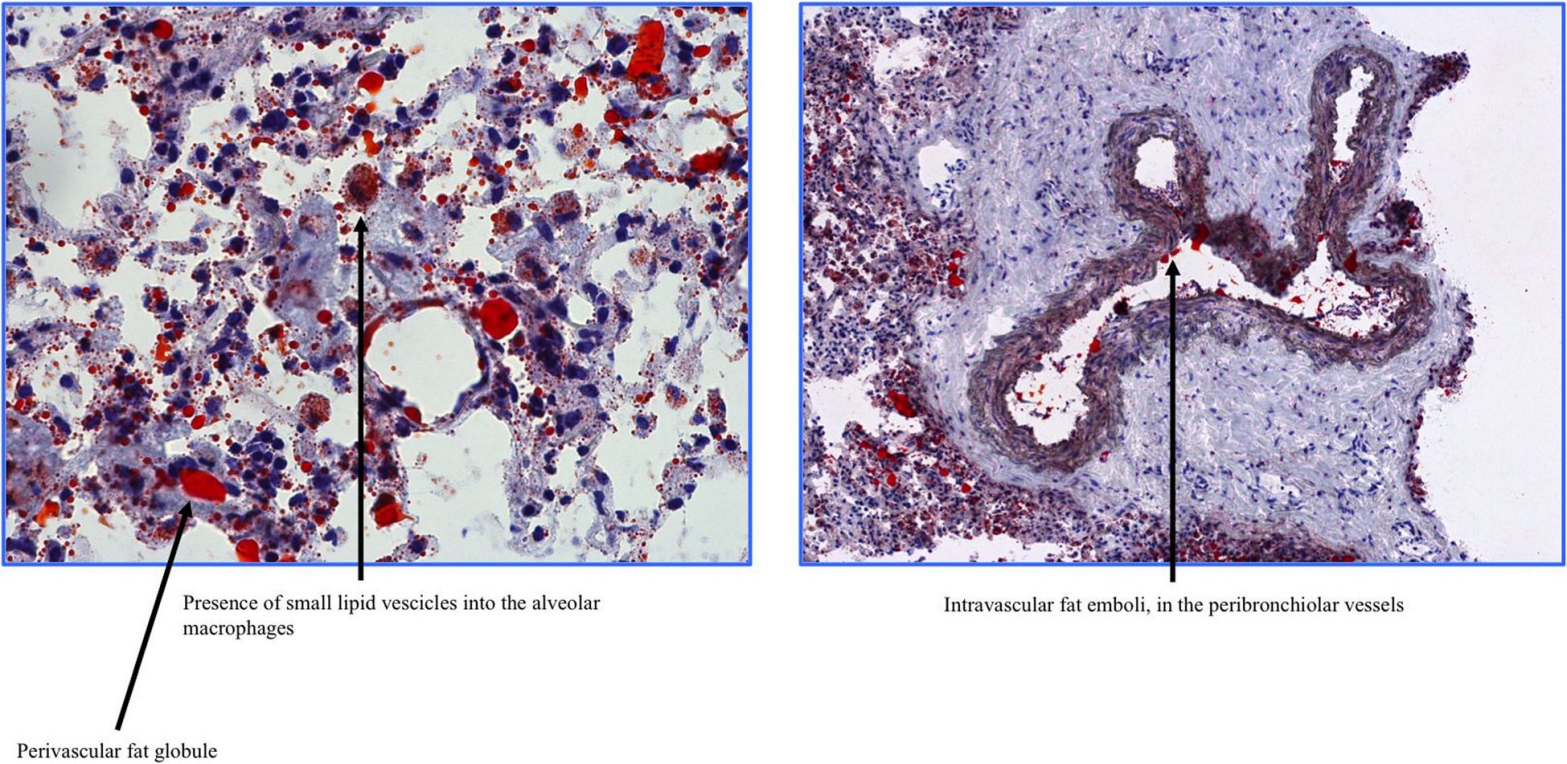
Oil Red O stained slides of the lung reveal extensive intravascular fat emboli within the peribronchiolar and alveolar vessels, as well as a perivascular fat globule and the presence of small lipid vesicles within alveolar macrophages

In the unfortunate event of death, macroscopic examinations of the lungs were found to reveal nonspecific areas of pulmonary infarction and hemorrhagic alveolitis, whereas microscopic examinations using Oil Red O staining revealed extensive intravascular FE in the peribronchiolar and alveolar vessels with small lipid vesicles located within alveolar macrophages (Fig. 2).

**Fig. 3:**
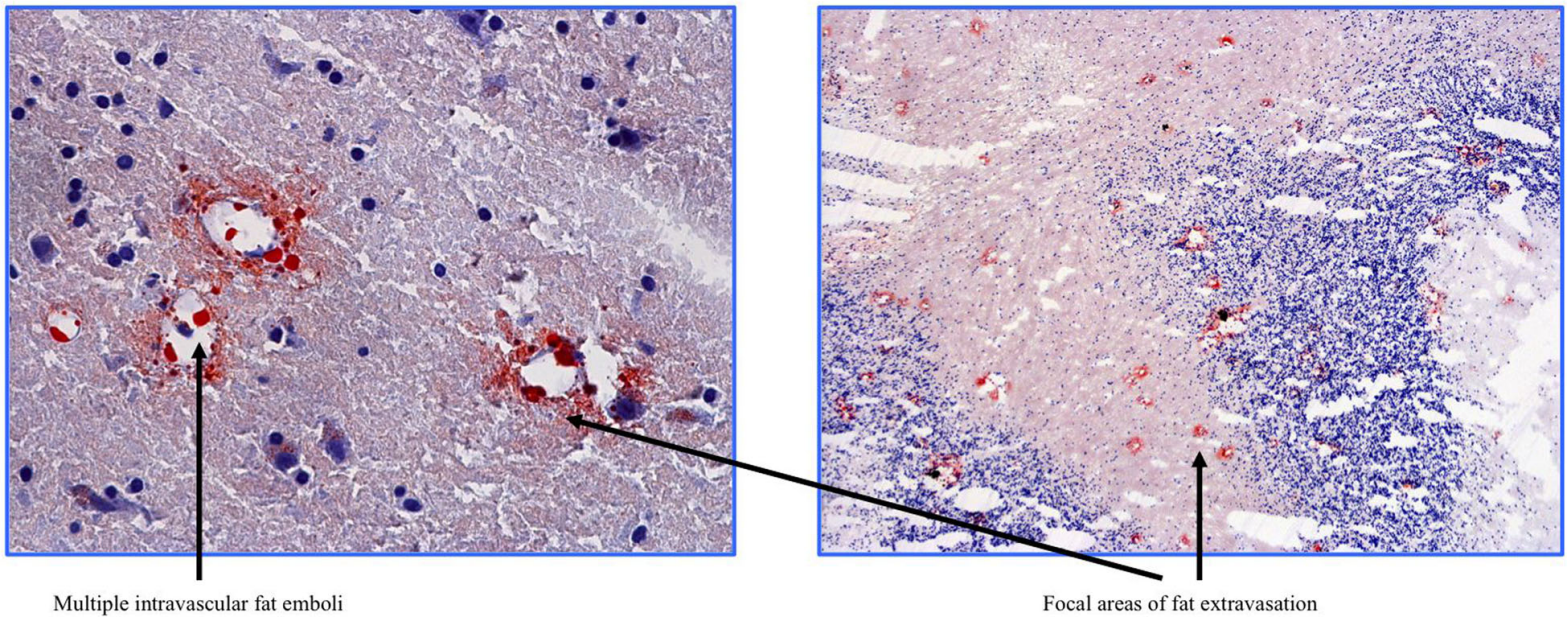
Multiple intravascular fat emboli and focal areas of fat extravasation in the central nervous system of a young patient whose cause of death was a massive cerebral fat embolism

**Fig. 4:**
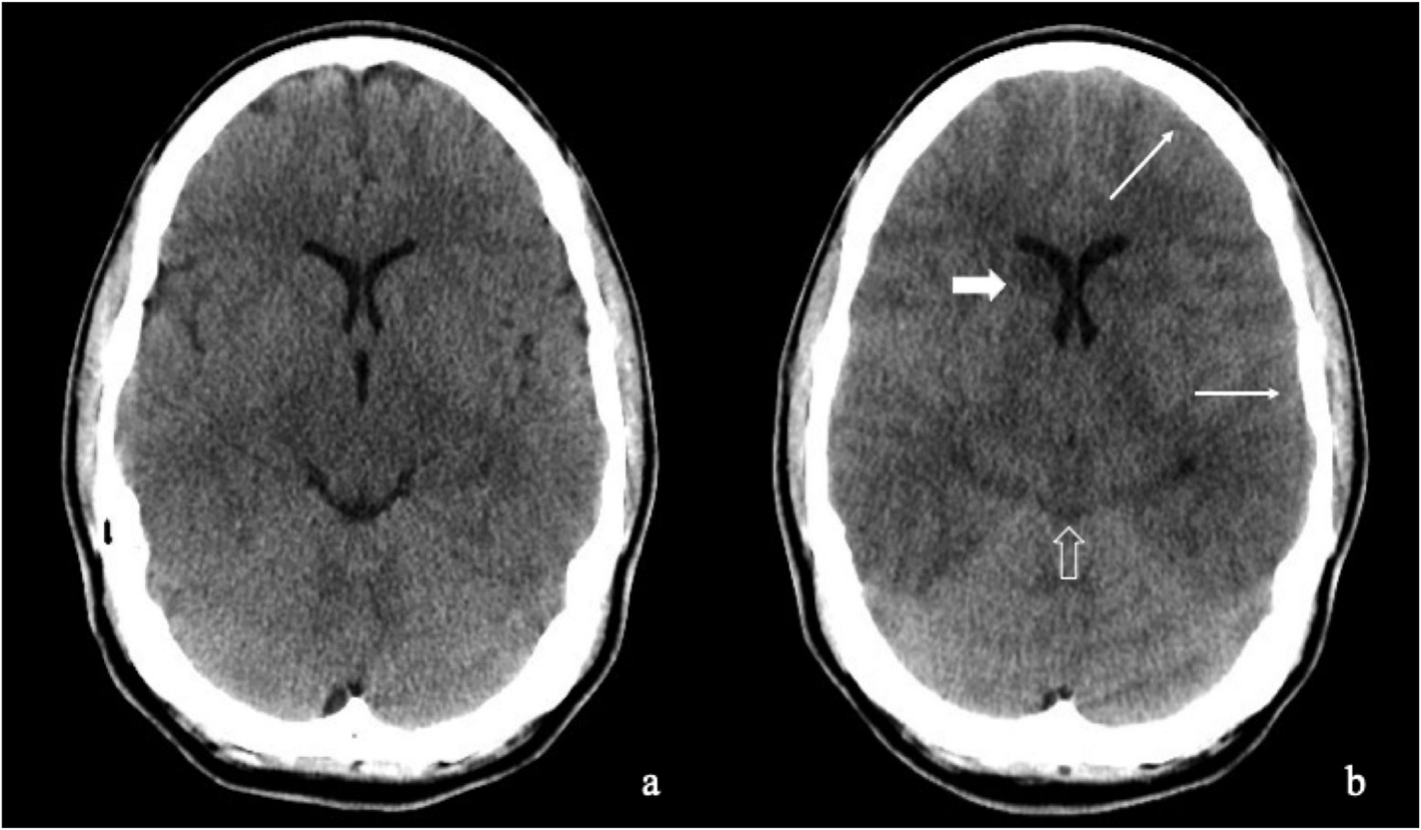
An axial non enhanced computed tomography (NECT) scan performed on the first day (**a**) and 24 h later (**b**) show the appearance of diffuse cerebral edema with initial loss of normal gray-white matter differentiation (narrow white arrows), obliteration of CSF spaces (unfilled think arrow) and the “disappearing” of the basal ganglia (i.e., head of the right caudate nucleus) (filled thick arrow)

Interest in a potential association between PFO and CFE as a putative mechanism of FES has been noted in the literature [38, 39]. Briefly, a PFO is the persistence into adulthood of an embryonic defect in the atrial septum in the form of a right to left cardiac shunt, and it has been proposed to form a possible anatomical link between venous thrombosis and acute (or sudden) stroke [24]. The data from the present study do not provide any evidence to support this. The prevalence of PFO at autopsy in the general population is approximately 27.3% [24]. Here, we found an incidence of just 12% – less than half that of the general population. If we were able to divide the cases according to the neurological consequences of FE/FES into ‘acute’ and ‘late’ cases, the presence of PFO might be able to explain the symptomatology of the former, whereas we might find PFO absent in the latter. The late manifestation may instead be related to pulmonary complications explained by a mechanism in which free fatty acids produce local biochemical mediators of oxidative stress that damage the capillary beds as a consequence of nitric oxide (NO), free radicals and inflammatory cytokines [36, 13]. In this event, microscopic examination of the brain would be expected to reveal perivascular cerebral oedema with areas of parenchymal and neuronal ischemic injury. Indeed, Oil Red O stained slides showed multiple intravascular fat emboli with focal areas of massive fat extravasation in patients with ‘late’ FES (Fig. 3), whereas brain CT scans of patients with ‘acute’ FES were reported to be normal. In one case, a massive cerebral oedema after acute FE was identified (Fig. 4).

With regard to central nervous system lesions, we found the prevalence CFE to be slightly higher in the basal ganglia, followed by the juxtacortical regions of the frontal, parietal and temporal lobes. It seems to reflect the different anatomical distribution of the vascular territories of the brain. In particular, there was a clear involvement of the so-called anterior circulation respect to the posterior circulation that includes the vertebrobasilar system supply, all the posterior fossa structures as well the midbrain, posterior thalami and the occipital lobes. No involvement of the posterior fossa structures was noted. It is interesting to highlight that, as in cardioembolic disease (of distinct origin), the brain’s involvement is more frequently bilateral, although involvement of the right side seems to be slightly more prevalent than the left in some cases. In general, intensive care support has markedly improved the outcome of patients with FES, decreasing the incidence of fatal cases from 15 to 20 to 7% [39].

In an elegant study, Byrick et al. presented their theory of the pathophysiology of FE in which they investigated the effects of FE in 21 anesthetized mongrel dogs without PFO following cemented arthroplasty [17]. They found that the deformability of the fatty material enabled it to pass through the lung filter under the appropriate pressure, forcing the liquefied fat into the open veins (this can explain why marrow fat needs to be forced into the veins to reach the lungs and then the head). We propose thinking about FES using the metaphor of a ‘test your strength’ fairground attraction hammer game, in which FES is an ‘upper-body syndrome’ related to high-velocity trauma. Moreover, since the fat globule is deformable and fluid, the pulmonary vascular occlusion constitutes a transient and variable phenomenon that may arise over a period, as shown in the Byrick canine study.

When a diagnosis of FES is suspected, a cerebral NMR must be performed, followed by supportive treatments focused on the clinical symptoms, and hemodynamic and respiratory support [40].

**Fig. 5:**
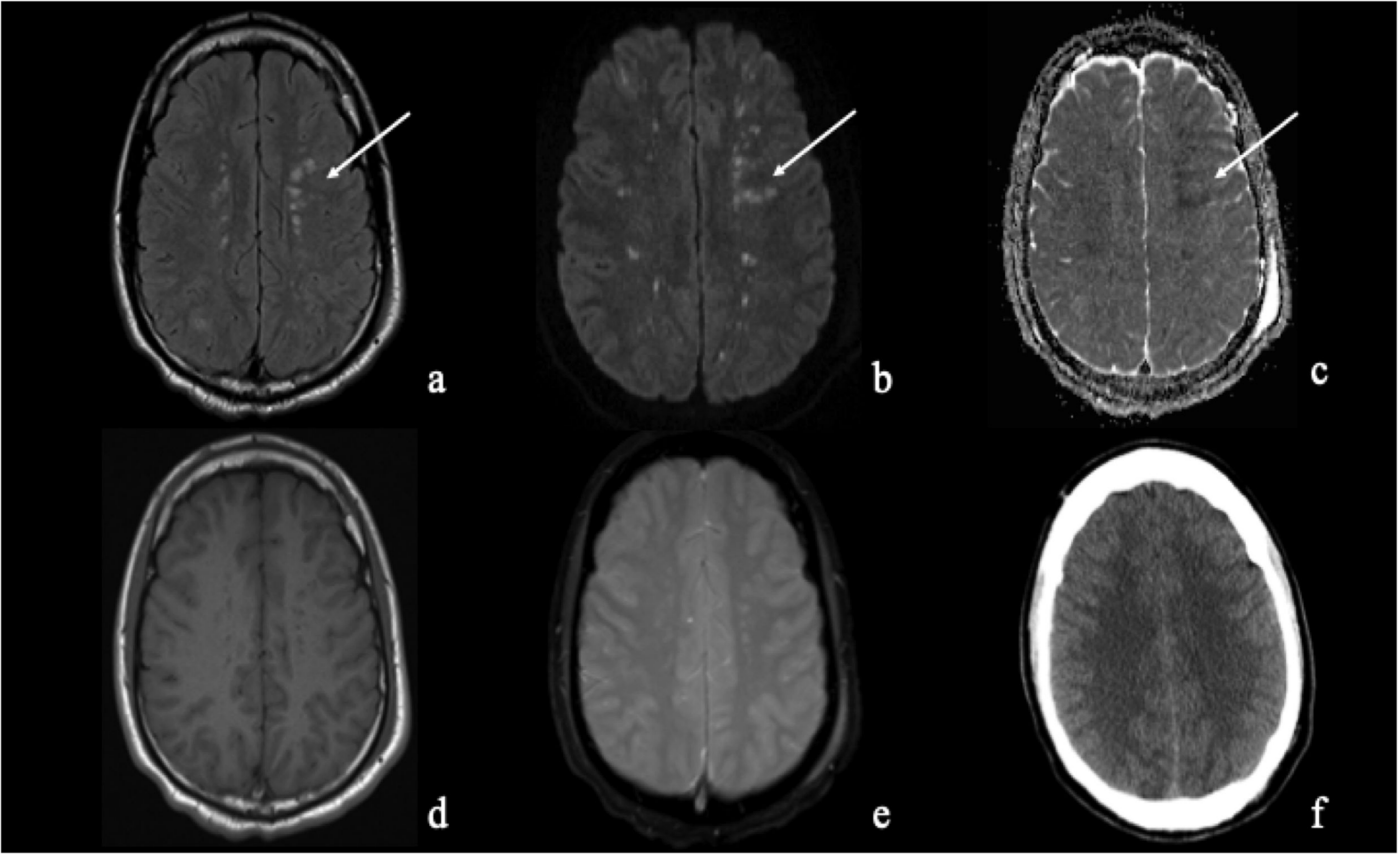
Axial FLAIR (**a**) magnetic resonance imaging sequence shows the presence of numerous multifocal hyperintensities involving the deep white matter of both hemispheres (arrow), which correspond to innumerable tiny foci of diffusion restrictions on DWI (**b**) with low signal on ADC map (**c**) (arrow). These findings are the so-called “starfield” pattern characteristic of cerebral fat embolism syndrome. In the same patient, the SE-T1W (**d**) and T2* GRE (**f**) sequences, and the axial non enhanced computed tomography NECT scan (**f**) appear relatively normal

Use of transcranial Doppler has also been suggested for the documentation of CFE [41, 42]. Figure S4 (supplementary material) shows a transcranial Doppler performed in a 19-year-old old boy that detected a microembolic signal in the left MCA following a saline/air injection. Near infrared spectroscopy (NIRS) or Bispectral Index (BIS) might also be available in an ICU and aid monitoring patient evolution. However, the most sensitive available exam remains MR imaging, which directly provides an anatomical correlate of the clinical severity of the brain injury (Fig. 5) [40]. In some case studies, MR imaging was able to show the disappearance of brain lesions after 2 months, associated with good clinical outcome [41–45].

At the time of this review, no established guidelines exist for the treatment of FE or FES and therapy remains largely supportive. One meta-analysis has suggested that corticosteroids might be beneficial in preventing FES and hypoxia without increasing the risk of infection, although mortality was unchanged [46].

The potential therapeutic role of heparin in FE remains unknown and its use in trauma patients is neither indicated nor contraindicated. In an ideal scenario, a diagnostic methodology or blood test specific for FES would be available. Of course, other pathologies are able to cause “non-traumatic” fat embolism (NTFE), such as sickle cell crisis, fatty liver disease, total parenteral nutrition, prolonged steroid use and non-traumatic joint arthroplasty [46, 47]. FE has been reported in the perinatal period around childbirth, although extremely rare [48].

In the last 10 years, the number of FES related published case reports has increased substantially [15, 16, 19–21]. The frequency of the disease has not undergone any significant changes, although it probably remains underdiagnosed in clinical practice.

The results of this structured literature analysis permitted us able to generate some hypotheses about fracture laterality, the presence of PFO and the underlying pathophysiological processes in FES that might help further our understanding of this pathology. Some limitations of the study also need to be acknowledged. Although we tried to include all case reports of adults with cerebral fat embolism published in the latest 58 years, in some of these reports, data were incomplete and as such not included in the study. Consequently, our results are based on the interpretation of the data analysis available from the detected cases.

## Conclusion

From this review of clinical cases published in the past 58 years, we can conclude that FE/FES is more frequent in men aged less than 30 years following multiple fractures of the legs. It appears that the laterality of the fracture is not related to the development of the syndrome. However, FES may be more frequent after a burst fracture in which the fat marrow is pushed to the upper region of the body through the lungs. Although most cases of FES occur 48–72 h after a fracture, further work is required to establish whether acute brain presentation (i.e., within 24 h) is related to the presence of PFO. Evidence of this could aid further research into obtaining an early diagnosis and guide a proactive approach to saving the brain from FE-induced lesions.

## Supplementary Information

**Additional file 1: Figure S1**. Study flow diagram according to the PRISMA reporting system.

**Additional file 2: Figure S2**. Bar chart showing the distribution of case reports according to patient gender and age.

**Additional file 3: Figure S3**. Case reports included in this review for each year since 1960.

**Additional file 4: Figure S4**. The figure above shows 3 MES detected in the left MCA after saline/air injection in a 19-year-old boy admitted to the Emergency Department following a car crash. At the medical examination, he was awake, cooperative and interacted regularly. Radiological findings demonstrated a femur fracture, a radius and ulna fracture, and a hip fracture, all of which were left-sided. After 36 h of hospital admission, the patient underwent surgical repair of the femur and upper arm fractures. Given increasing drowsiness and hypoxemia after the operation, the patient was admitted to the ICU. A neuro diffusion-weighted MRI showed the classic “starfield” appearance of multiple foci of restricted diffusion with circled areas – pathognomonic evidence of cerebral fat embolism. The patient did not require endotracheal intubation or mechanical ventilation. After 72 h, he was transferred to the Neurology Department, where his clinical condition improved to the point of being discharged.

**Additional file 5: Table S1**. Key characteristics of case reports. **Table S2**. Main differences between men and women. **Table S3**. Fracture site. **Table S4**. Fracture laterality in men vs. women. **Table S5**. Type and laterality of fracture per age category. **Table S6**. Characteristics of brain lesions identified by neuroimaging. **Table S7**. Location of central nervous system involvement according to gender. **Table S8**. Percentage of cases per age category positive for PFO and fat emboli as ascertained by brain imaging. **Table S9**. Anatomic distribution of cerebral lesions per age category. **Table S10**. Univariate and multivariate logistic regression for assessing risk factors with increasing age. **Table S11**. Univariate and multivariate logistic regression for assessing risk factors in woman vs. men with fat cerebral embolism.

## Data Availability

Data are available following a reasoned request.
