# Supplementary material for: Cerebral fat embolism after traumatic bone fractures: a structured literature review and analysis of published case reports
Source: Scand J Trauma Resusc Emerg Med. 2021 Mar 12;29:47. doi: 10.1186/s13049-021-00861-x (PMC7953582; doi:10.1186/s13049-021-00861-x)
Supplement: Supplementary file 1 — Additional file 1: Figure S1. Study flow diagram according to the PRISMA reporting system. [file 13049_2021_861_MOESM1_ESM.pdf]

PRISMA 2009 Flow Diagram

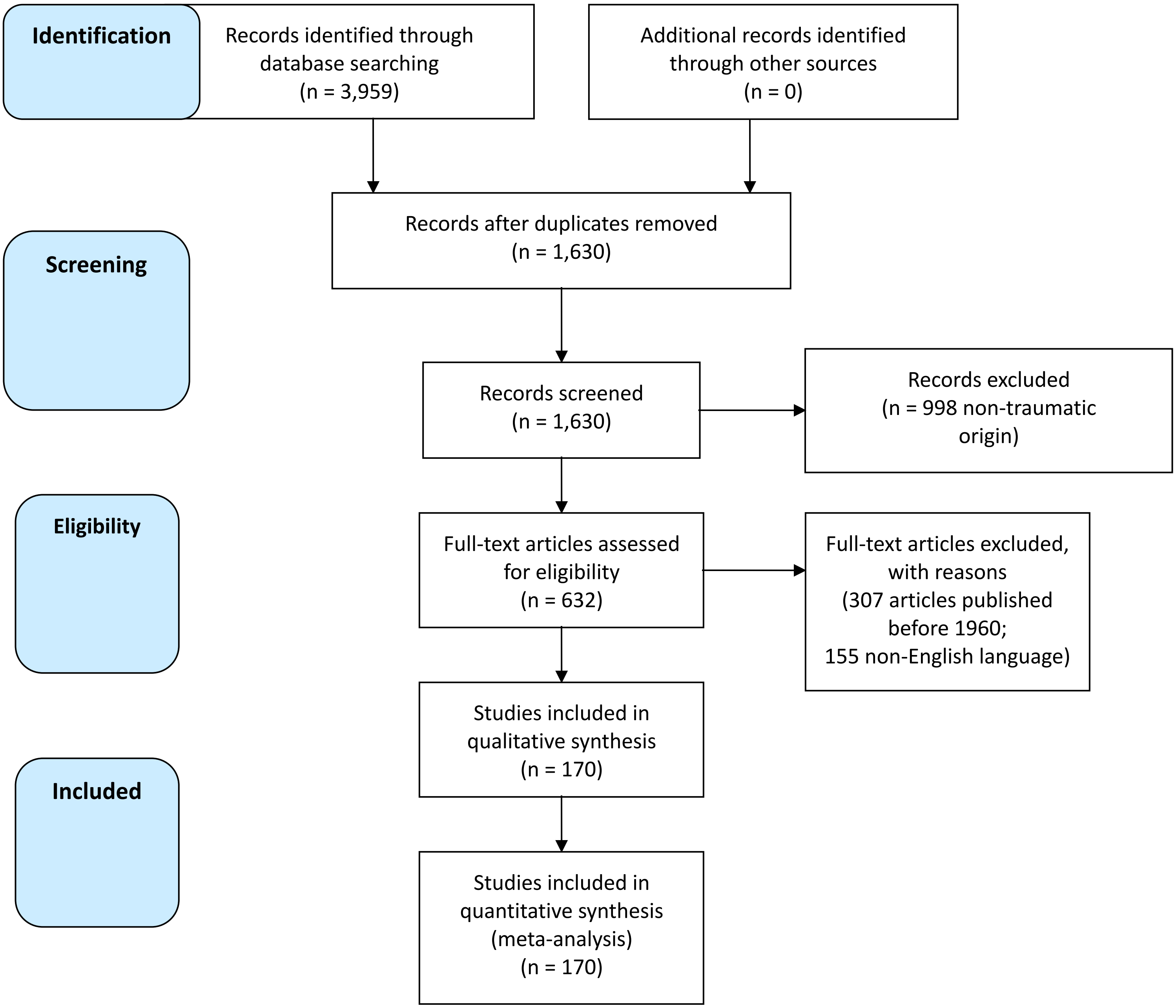

From: Moher D, Liberati A, Tetzlaff J, Altman DG, The PRISMA Group (2009). *P*referred *R*eporting *I*tems for *S*ystematic Reviews and *M*eta-*A*nalyses: The PRISMA Statement. PLoS Med 6(7): e1000097. doi:10.1371/journal.pmed1000097

For more information, visit www.prisma-statement.org.
