# Supplementary figures and images for: Cerebral fat embolism after traumatic bone fractures: a structured literature review and analysis of published case reports
Source: Scand J Trauma Resusc Emerg Med. 2021 Mar 12;29:47. doi: 10.1186/s13049-021-00861-x (PMC7953582; doi:10.1186/s13049-021-00861-x)

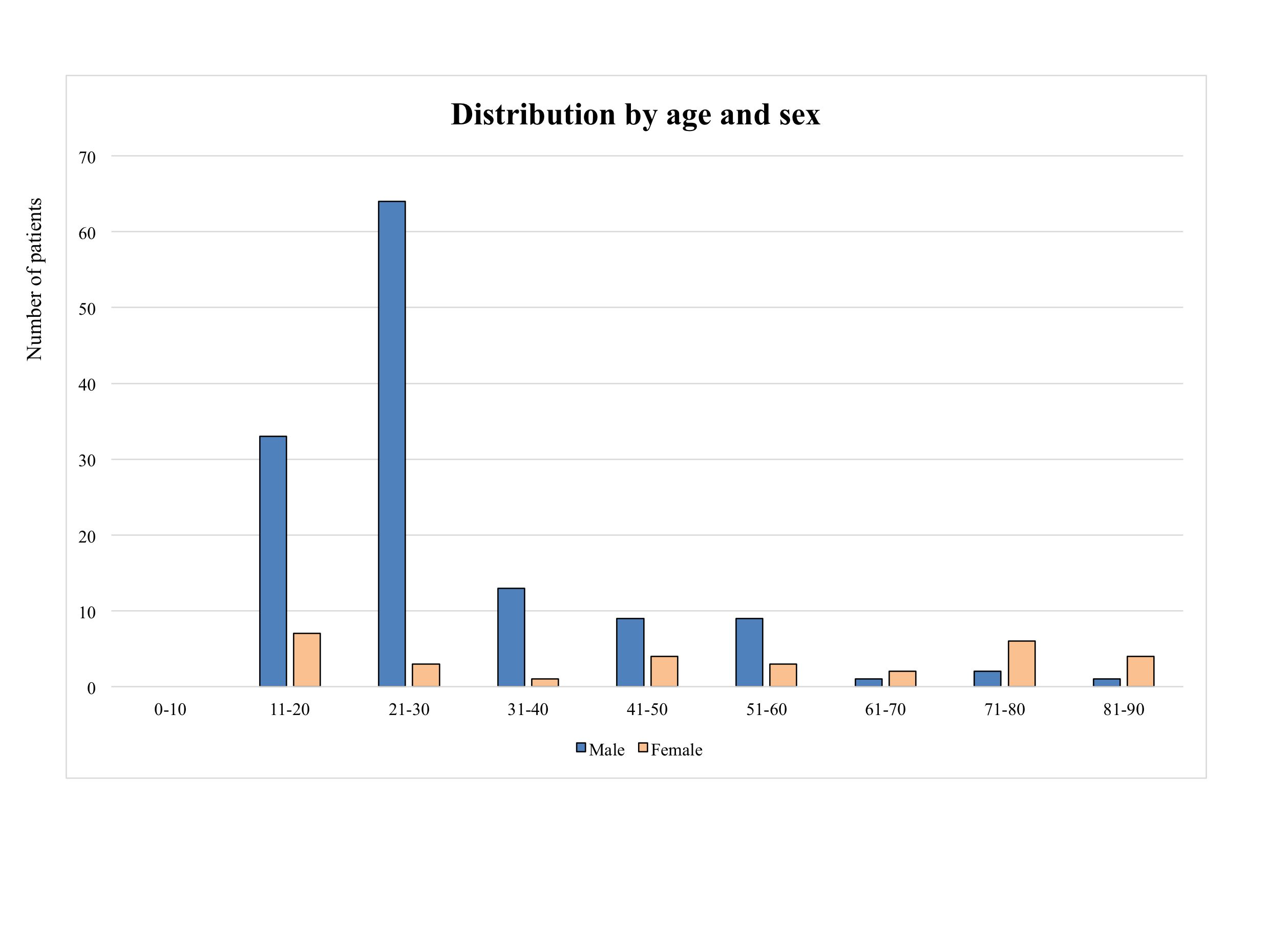

Supplement: Supplementary file 2 — Additional file 2: Figure S2. Bar chart showing the distribution of case reports according to patient gender and age. [file 13049_2021_861_MOESM2_ESM.jpg]

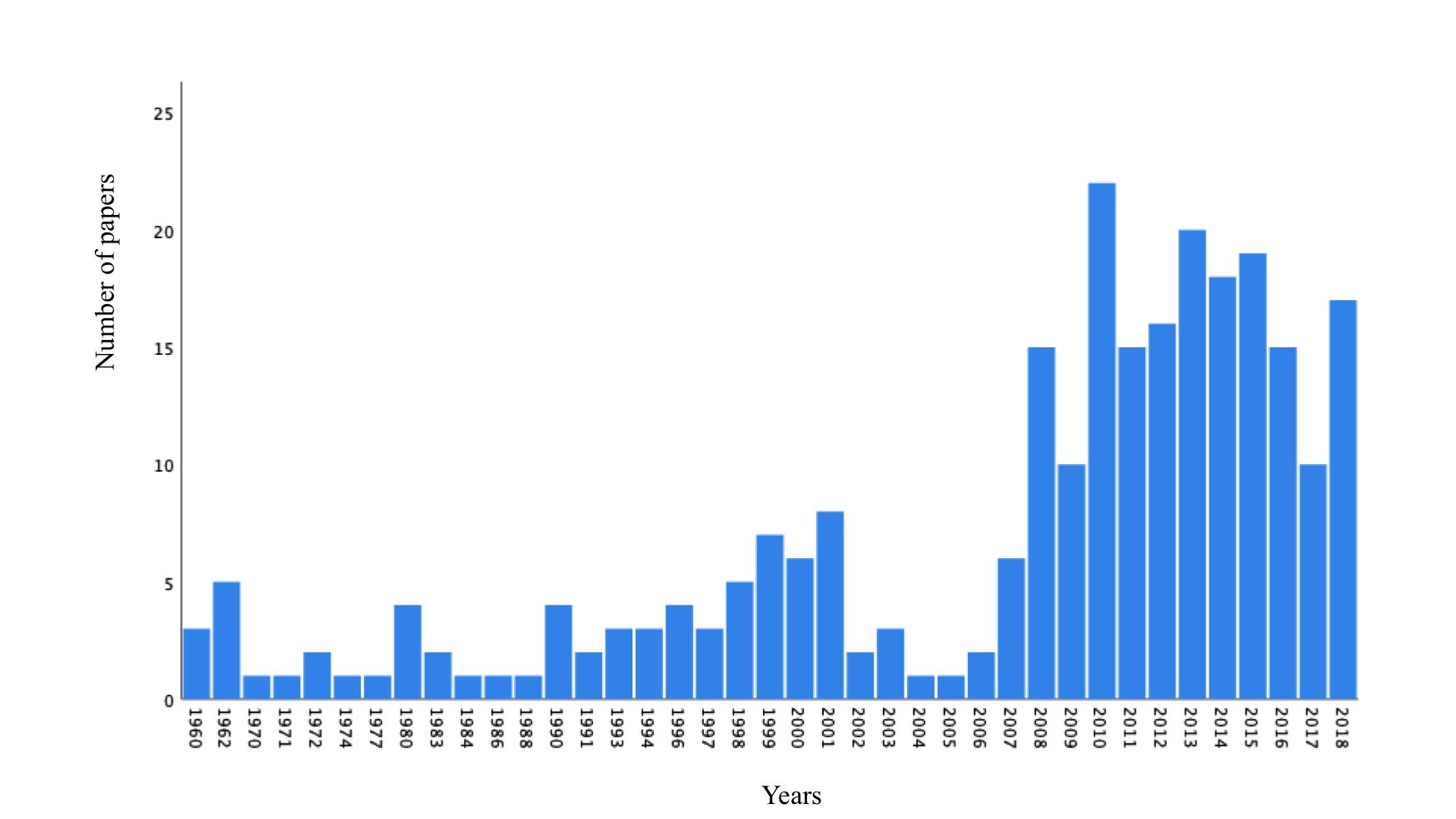

Supplement: Supplementary file 3 — Additional file 3: Figure S3. Case reports included in this review for each year since 1960. [file 13049_2021_861_MOESM3_ESM.jpg]

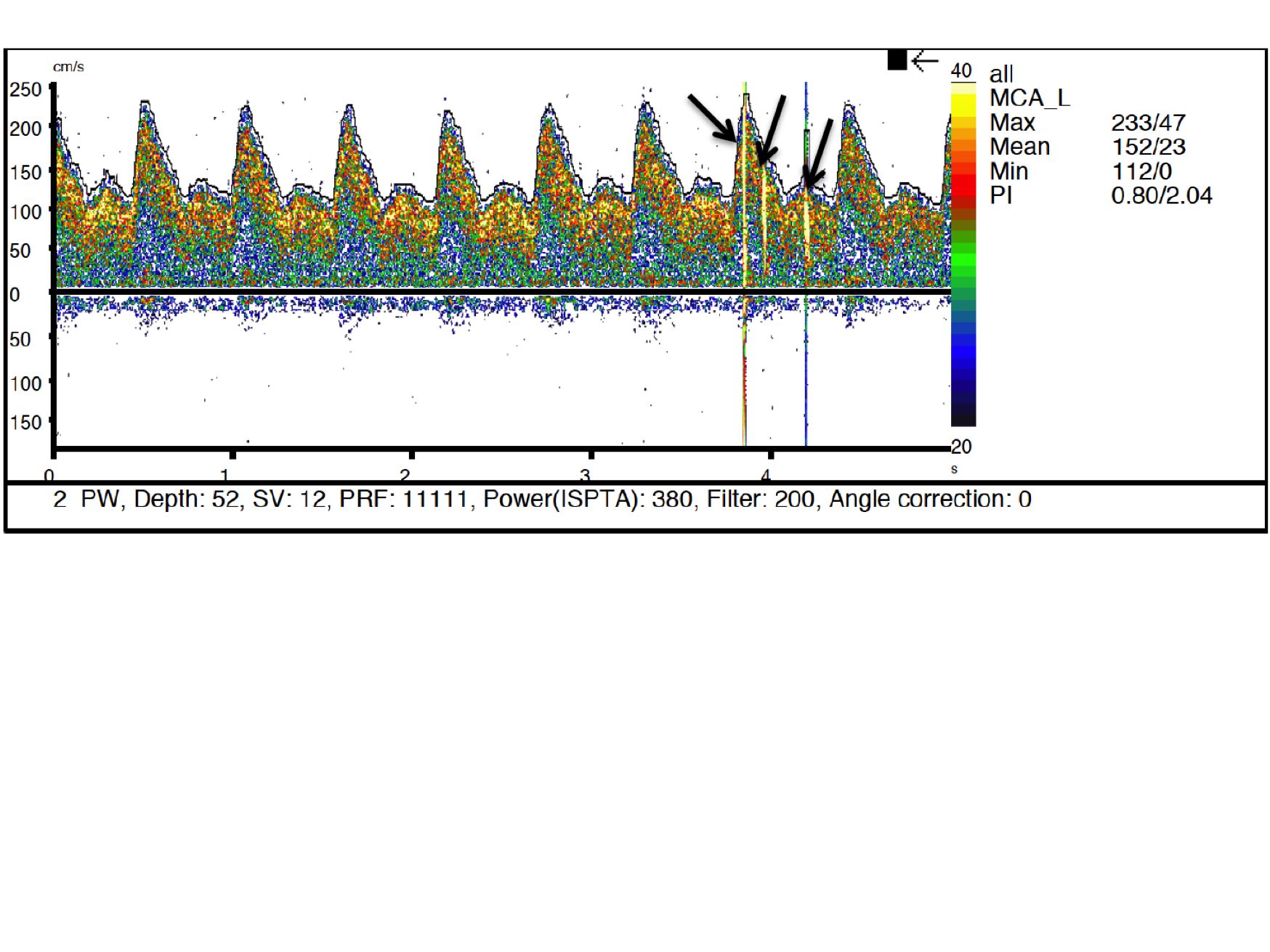

Supplement: Supplementary file 4 — Additional file 4: Figure S4. The figure above shows 3 MES detected in the left MCA after saline/air injection in a 19-year-old boy admitted to the Emergency Department following a car crash. At the medical examination, he was awake, cooperative and interacted regularly. Radiological findings demonstrated a femur fracture, a radius and ulna fracture, and a hip fracture, all of which were left-sided. After 36 h of hospital admission, the patient underwent surgical repair of the femur and upper arm fractures. Given increasing drowsiness and hypoxemia after the operation, the patient was admitted to the ICU. A neuro diffusion-weighted MRI showed the classic “starfield” appearance of multiple foci of restricted diffusion with circled areas – pathognomonic evidence of cerebral fat embolism. The patient did not require endotracheal intubation or mechanical ventilation. After 72 h, he was transferred to the Neurology Department, where his clinical condition improved to the point of being discharged. [file 13049_2021_861_MOESM4_ESM.jpg]
