# Supplementary material for: Cerebral fat embolism after traumatic bone fractures: a structured literature review and analysis of published case reports
Source: Scand J Trauma Resusc Emerg Med. 2021 Mar 12;29:47. doi: 10.1186/s13049-021-00861-x (PMC7953582; doi:10.1186/s13049-021-00861-x)
Supplement: Supplementary file 5 — Additional file 5: Table S1. Key characteristics of case reports. Table S2. Main differences between men and women. Table S3. Fracture site. Table S4. Fracture laterality in men vs. women. Table S5. Type and laterality of fracture per age category. Table S6. Characteristics of brain lesions identified by neuroimaging. Table S7. Location of central nervous system involvement according to gender. Table S8. Percentage of cases per age category positive for PFO and fat emboli as ascertained by brain imaging. Table S9. Anatomic distribution of cerebral lesions per age category. Table S10. Univariate and multivariate logistic regression for assessing risk factors with increasing age. Table S11. Univariate and multivariate logistic regression for assessing risk factors in woman vs. men with fat cerebral embolism. [file 13049_2021_861_MOESM5_ESM.docx]

| Number of patients | 268 |
| --- | --- |
| Sex ratio (M/F) (%) | 81.6/18.4 |
| Mean age in years (±SD) | 33.15 (±18.34) |
| Presence of foramen ovale pervium (%) | 11.6 |
| CT brain scan available (%) | 73.4 |
| Brain MRI available (%) | 77.7 |

Table S1. Key characteristics of case reports

Legend: computed tomography (CT); magnetic resonance imaging (MRI).

|  | Male | Female | p |
| --- | --- | --- | --- |
| Mean age in years (±SD) | 29 (14) | 51 (26) | <0.001 |
| Foramen ovale pervium (%) | 8.5 | 5.6 | 0.478 |
| CT brain scan positive for CFE (%) | 53.8 | 17.2 | 0.006 |
| Brain MRI positive for CFE (%) | 54.1 | 23.8 | 0.874 |

Table S2. Main differences between men and women.

Legend: computed tomography (CT); magnetic resonance imaging (MRI).

cerebral fat embolism (CFE).

| Site of bone fractures (%) | |
| --- | --- |
| Femoral fracture | 70.0 |
| Tibial fracture | 36.6 |
| Peroneum fracture | 19.1 |
| Ulnar fracture | 3.4 |
| Radium fracture | 4.8 |
| Humerus fracture | 3.4 |
| Ribs fracture | 4.9 |
| Clavicular fracture | 2.6 |
| Pelvic fracture | 7.6 |
| Multiple fractures of upper bones | 2.6 |
| Multiple fractures of lower bones | 37.3 |
| Multiple fracture of upper and lower bones | 13.4 |
| Right side fractures | 41.6 |
| Left side fractures | 27 |
| Bilateral fractures | 31.5 |

Table S3: Fracture site

| Fracture site | Male (%) | Female (%) | p |
| --- | --- | --- | --- |
| Femoral fracture | 48.2 | 19.7 | 0.364 |
| Tibial fracture | 25.4 | 10.4 | 0.101 |
| Peroneum fracture | 14 | 5.1 | 0.061 |
| Ulnar fracture | 2.1 | 0 | 0.173 |
| Radium fracture | 0.5 | 1 | 0.084 |
| Homerus fracture | 3.1 | 0 | 0.194 |
| Rib fracture | 3.6 | 3.1 | 0.279 |
| Clavicular fracture | 1.6 | 0 | 0.241 |
| Pelvic fracture | 4.7 | 2.1 | 0.979 |
| Multiple fractures of upper bones  Multiple fractures of lower bones  Multiple fractures of upper and lower bones | 1.6  23.8  9.3 | 1  9.3  7.3 | 0.364 |
| Right side fractures  Left side fractures  Bilateral fractures | 29.7  21.4  17.7 | 17.7  4.2  9.4 | 0.031 |

Table S4. Fracture laterality in men vs. women.

| Fracture site | Age groups | | | | | | | P |
| --- | --- | --- | --- | --- | --- | --- | --- | --- |
|  | ≤ 18 | 19-30 | 31-40 | 41-50 | 51-60 | 61-70 | ≥ 70 |  |
| Femoral fracture | 1.7 | 26.5 | 8.5 | 7 | 9.8 | 2.6 | 15.2 | 0.003 |
| Tibial fracture | 1.5 | 14.3 | 3.3 | 2.6 | 5.4 | 1.3 | 7.6 | 0.840 |
| Peroneum fracture | 0.9 | 7 | 2.6 | 1.7 | 3.3 | 1.3 | 3 | 0.272 |
| Ulnar fracture | 0 | 1.7 | 0 | 0 | 0 | 0 | 0 | 0.047 |
| Radium fracture | 0.2 | 3 | 0.7 | 0 | 0 | 0 | 0 | 0.008 |
| Homerus fracture | 0.4 | 0.9 | 0.7 | 0.9 | 1.1 | 0 | 0 | 0.023 |
| Ribs fracture | 0.2 | 0.9 | 0.7 | 0.9 | 2.2 | 0 | 3 | 0.003 |
| Clavicular fracture | 0 | 0.4 | 0 | 0 | 2.2 | 0 | 0 | 0.000 |
| Pelvic fracture | 0.2 | 2.2 | 1.3 | 0.9 | 1.1 | 0 | 1.5 | 0.482 |
| Multiple fractures of upper bones  Multiple fractures of lower bones  Multiple fracture of upper and lower bones | 0.2  1.3  0.0 | 0.4  10.9  7.4 | 0.7  5.2  0 | 0  5.2  0 | 1.1  5.4  3.3 | 0  1.3  2.6 | 0  6.1  4.6 | 0.000 |
| Right side fractures  Left side fractures  Bilateral fractures | 1.1  0.4  2.2 | 18.9  12.3  8.3 | 5.3  1.3  2.6 | 6.1  2.6  1.8 | 4.4  6.6  2.2 | 2.6  0  1.3 | 7.7  4.6  7.7 | 0.012 |

Table S5. Type and laterality of fracture per age category.

| Brain lesion characteristics | |
| --- | --- |
| Frontal lobe:   - No involvement - Right - Left - Bilateral | 68.3%  1.6%  4.8%  25.4% |
| Parietal lobe:   - No involvement - Right - Left - Bilateral | 74.6%  0  3.2%  22.2% |
| Temporal lobe:   - No involvement - Right - Left - Bilateral | 81.8%  1.8%  0  16.4% |
| Occipital lobe:   - No involvement - Right - Left - Bilateral | 82.5%  1.8%  1.8%  14% |
| Internal Capsule   - No involvement - Right - Left - Bilateral | 17.6%  1.5%  1.5%  79.4% |
| Subcortical   - No involvement - Right - Left - Bilateral | 11%  1.2%  2.4%  85.4% |
| Involvement of ≥ 3 brain areas   - Right - Left | 7.3%  4.9% |

Table S6. Characteristics of brain lesions identified by neuroimaging

|  | Male (%) | Female (%) | p |
| --- | --- | --- | --- |
| Frontal lobe:   - No involvement - Right - Left - Bilateral | 58.8  1.5  2.9  22.1 | 8.8  0  2.9  2.9 | 0.225 |
| Parietal lobe:   - No involvement - Right - Left - Bilateral | 65.7  0  3  19.4 | 9  0  0  3 | 0.861 |
| Temporal lobe:   - No involvement - Right - Left - Bilateral | 70  0  0  13.3 | 10  3.3  0  3.3 | 0.005 |
| Occipital lobe:   - No involvement - Right - Left - Bilateral | 72.1  1.6  1.6  11.5 | 9.8  0  0  3.3 | 0.798 |
| Internal Capsule   - No involvement - Right - Left - Bilateral | 16  1.3  1.3  62.7 | 0  0  0  18.7 | 0.267 |
| Subcortical   - No involvement - Right - Left - Bilateral | 9.9  1.1  1.1  68.1 | 0  0  2.2  17.6 | 0.084 |
| Involvement of ≥ 3 brain areas   - Right - Left | 4.4  4.4 | 4.4  0 | 0.180 |

TABLE S7. Location of central nervous system involvement according to gender.

|  | ≤ 18 | 19-30 | 31-40 | 41-50 | 51-60 | 61-70 | ≥ 70 | P |
| --- | --- | --- | --- | --- | --- | --- | --- | --- |
| M/F (%) | 2.6/1.1 | 37/2.2 | 8.5/0.7 | 7.8/3.5 | 9.8/3.3 | 1.3/2.6 | 4.6/15.2 | 0.000 |
| Patent foramen ovale (%) | 0.8 | 5.8 | 0 | 0 | 0 | 0 | 8.7 | 0.000 |
| Positive CT brain scan (%) | 3 | 32.7 | 7.5 | 5.5 | 6.8 | 2.7 | 9.5 | 0.000 |
| Positive brain MRI (%) | 2.7 | 32.4 | 6.8 | 7.3 | 12.6 | 2.7 | 11.2 | 0.000 |

Table S8. Percentage of cases per age category positive for PFO and fat emboli as ascertained by brain imaging.

|  | ≤ 18 | 19-30 | 31-40 | 41-50 | 51-60 | 61-70 | ≥ 70 | P |
| --- | --- | --- | --- | --- | --- | --- | --- | --- |
| Frontal lobe:   - No involvement - Right - Left - Bilateral | 1.9  0  0.6  1.9 | 37.4  1.3  1.3  12.9 | 3.9  0  0  1.9 | 7.7  0  0  0 | 9.7  0  3.2  3.2 | 3.9  0  0  0 | 4.5  0  0  4.5 | 0.034 |
| Parietal lobe:   - No involvement - Right - Left - Bilateral | 3.9  0  0.7  0 | 39.2  0  1.3  13.1 | 3.9  0  0  3.9 | 7.8  0  0  7.8 | 9.8  0  0  3.3 | 3.9  0  0  0 | 4.6  0  0  4.6 | 0.020 |
| Temporal lobe:   - No involvement - Right - Left - Bilateral | 3.5  0  0  0 | 44  0  0  5.7 | 4.3  0  0  2.1 | 2.8  0  0  5.7 | 10.6  3.5  0  3.5 | 4.3  0  0  0 | 5  0  0  5 | 0.000 |
| Occipital lobe:   - No involvement - Right - Left - Bilateral | 3.5  0.7  0.7  0 | 44.8  0  0  4.2 | 4.2  0  0  2.1 | 5.6  0  0  2.8 | 10.5  0  0  7 | 4.2  0  0  0 | 4.9  0  0  4.9 | 0.001 |
| Internal Capsule   - No involvement - Right - Left - Bilateral | 0.6  0  0  4.1 | 12.9  1.2  0  35.3 | 0  0  1.8  7.1 | 0  0  0  9.4 | 0  0  0  11.8 | 0  0  0  3.5 | 0  0  0  12.4 | 0.001 |
| Subcortical   - No involvement - Right - Left - Bilateral | 25  0  0  75 | 11.1  1.9  1.9  85.2 | 0  0  0  100 | 0  0  0  100 | 16.7  0  0  83.7 | 0  0  0  100 | 0  0  25  75 | 0.230 |
| Involvement of ≥ 3 brain territory   - No involvement - Right - Left | 2  1  1 | 51  2  0 | 5.9  0  2.9 | 0  0  0 | 14.7  0  0 | 5.9  0  0 | 6.9  6.9  0 | 0.000 |

Table S9. Anatomic distribution of cerebral lesions per age category.

|  | Univariate | | Multivariate | |
| --- | --- | --- | --- | --- |
|  | RR (95% CI) | p | RR (95% CI) | p |
| Sex | 1.874 (1.272-2.477) | 0.000 | 1.412 (0.484-2.341) | 0.000 |
| Ribs fracture | 0.202 (0.015-0.388) | 0.034 | - | - |

Table S10. Univariate and multivariate logistic regression for assessing risk factors with increasing age

|  | Univariate | | Multivariate | |
| --- | --- | --- | --- | --- |
|  | RR (95% CI) | p | RR (95% CI) | p |
| Age | 1.054 (1.036-1.07) | 0.000 | 1.048 (1.029-1.066) | 0.000 |
| Positive brain CT scan | 0.4 (0.205-0.780) | 0.007 | - | - |
| Sides of fractures | 0.876 (0.606-1.265) | 0.480 | - | - |
| Involvement of temporal lobes | 1.254 (0.728-2.158) | 0.414 | -- | - |

Table S11. Univariate and multivariate logistic regression for assessing risk factors in woman vs. men with fat cerebral embolism
